# The expression pattern of GDF15 in human brain changes during aging and in Alzheimer’s disease

**DOI:** 10.3389/fnagi.2022.1058665

**Published:** 2023-01-09

**Authors:** Antonio Chiariello, Sabrina Valente, Gianandrea Pasquinelli, Alessandra Baracca, Gianluca Sgarbi, Giancarlo Solaini, Valentina Medici, Valentina Fantini, Tino Emanuele Poloni, Monica Tognocchi, Marina Arcaro, Daniela Galimberti, Claudio Franceschi, Miriam Capri, Stefano Salvioli, Maria Conte

**Affiliations:** ^1^Department of Experimental, Diagnostic and Specialty Medicine (DIMES), University of Bologna, Bologna, Italy; ^2^Department of Biomedical and Neuromotor Sciences (DIBINEM), Laboratory of Biochemistry and Mitochondrial Pathophysiology, University of Bologna, Bologna, Italy; ^3^Department of Neurology and Neuropathology, Golgi-Cenci Foundation, Milan, Italy; ^4^Department of Agriculture, Food and Environment, University of Pisa, Pisa, Italy; ^5^Fondazione Ca’ Granda IRCCS Ospedale Maggiore Policlinico, Milan, Italy; ^6^Department of Applied Mathematics of the Institute of ITMM, National Research Lobachevsky State University of Nizhny Novgorod, Nizhny Novgorod, Russia; ^7^Interdepartmental Centre “Alma Mater Research Institute on Global Challenges and Climate Change (Alma Climate)”, University of Bologna, Bologna, Italy

**Keywords:** GDF15, Alzheimer’s disease, aging, inflammation, mitochondrial dysfunction

## Abstract

**Introduction:**

Growth Differentiation Factor 15 (GDF15) is a mitochondrial-stress-responsive molecule whose expression strongly increases with aging and age-related diseases. However, its role in neurodegenerative diseases, including Alzheimer’s disease (AD), is still debated.

**Methods:**

We have characterized the expression of GDF15 in brain samples from AD patients and non-demented subjects (controls) of different ages.

**Results:**

Although no difference in CSF levels of GDF15 was found between AD patients and controls, GDF15 was expressed in different brain areas and seems to be predominantly localized in neurons. The ratio between its mature and precursor form was higher in the frontal cortex of AD patients compared to age-matched controls (*p* < 0.05). Moreover, this ratio was even higher for centenarians (*p* < 0.01), indicating that aging also affects GDF15 expression and maturation. A lower expression of OXPHOS complexes I, III, and V in AD patients compared to controls was also noticed, and a positive correlation between *GDF15* and *IL-6* mRNA levels was observed. Finally, when GDF15 was silenced *in vitro* in dermal fibroblasts, a decrease in OXPHOS complexes transcript levels and an increase in *IL-6* levels were observed.

**Discussion:**

Although GDF15 seems not to be a reliable CSF marker for AD, it is highly expressed in aging and AD brains, likely as a part of stress response aimed at counteracting mitochondrial dysfunction and neuroinflammation.

## Introduction

Alzheimer’s disease (AD) is the most common neurodegenerative disorder. It is characterized by a progressive loss of neurons and synaptic connections, deposition of amyloid-beta (Aβ) plaques, and neurofibrillary tangles of phosphorylated Tau protein (Pradeepkiran and Reddy, 2020; Wang et al., 2020). The first brain areas affected by the disease are the entorhinal cortex and the hippocampus, then the alterations spread to the parietal, temporal, and frontal lobes. In particular, the spreading follows a typical distribution across the various regions of the cerebral cortex outlined by the Braak stages (Gómez-Isla et al., 1996; Braak et al., 2006; Wang et al., 2010). The mechanisms that drive these phenomena are not totally clarified and include inflammation, oxidative stress, and mitochondrial dysfunction (Bosetti et al., 2002; Pradeepkiran and Reddy, 2020; Wang et al., 2020). It is known that mitochondrial dysfunction can elicit a complex stress response, including mitochondrial unfolded protein response (UPR^mt^) and endoplasmic reticulum stress response (Lim et al., 2009; Rose et al., 2017). Within the downstream effects of these stress responses, there is the production of Growth Differentiation Factor 15 (GDF15; Yang et al., 2010; Melber and Haynes, 2018). GDF15, also known as macrophage inhibitory cytokine 1 (MIC-1), placental transformation growth factor (PTGF-b), prostate derived factor (PDF), placental bone morphogenetic protein (PLAB), and NSAID activated gene-1 (NAG-1), is a distant member of the transforming growth factor-β (TGF-β) superfamily (Bootcov et al., 1997). It is also considered a “mitokine,” i.e., a soluble molecule produced and secreted in response to mitochondrial stress and able to induce an adaptive response also in distant cells not directly affected by the stressful agent (Durieux et al., 2011; Conte et al., 2020a).

The expression of GDF15 occurs in particular at the level of reproductive tissues, placenta, bladder, skeletal muscle, and liver. It is regulated by several transcriptional factors, including Activating Transcription Factor 3 and 4 (ATF3 and ATF4), DNA damage inducible transcript 3 (DDIT3), and p53 (Osada et al., 2007; Kelly et al., 2009; Costa-Mattioli and Walter, 2020; Lockhart et al., 2020; Kang et al., 2021; Conte et al., 2022). GDF15 is first synthetized as a precursor protein (pro-GDF15) of 308 amino acids that undergoes a disulfide bond-mediated dimerization and is then cleaved into the mature form (m-GDF15) of 112 amino acids. To date, m-GDF15 is considered the most biologically active form of the protein, which is secreted from the cell through still unknown secretory pathways (Li et al., 2018; Conte et al., 2022). At present, the best-characterized biological mechanism of action of GDF15 is through GDNF α-like receptor (GFRAL), whose expression seems to be limited to the area postrema and the nucleus of the solitary tract, two areas of the hindbrain (Emmerson et al., 2017; Hsu et al., 2017; Mullican et al., 2017; Yang et al., 2017). To date, however, the precise biological functions of GDF15 are still not fully clarified. Studies have shown that it plays a central role as a regulator of appetite, body weight, and energy balance. GDF15 can induce nausea and is recognized as a key mediator of cancer-associated cachexia (Patel et al., 2019; Suriben et al., 2020; Conte et al., 2022). Moreover, GDF15 can exert anti-inflammatory activity by regulating tissue tolerance to inflammation and infections and by reducing the expression of pro-inflammatory cytokines (Bootcov et al., 1997; Moon et al., 2020; Conte et al., 2020a, 2022).

Interestingly, GDF15 has emerged as one of the most upregulated proteins during aging (Tanaka et al., 2018; Conte et al., 2019; Lehallier et al., 2019; Conte et al., 2020b;), and it has been proposed as a biomarker of aging (Liu et al., 2021; Shen et al., 2022). Moreover, high circulating levels of GDF15 were positively associated with different age-related diseases, such as cardiovascular diseases, type 2 diabetes, and sarcopenia (Andersson et al., 2020; Kim et al., 2020; Conte et al., 2021, 2022), although its role in these pathologies and in the aging process is not yet clear. Several studies in humans have found an association between high circulating levels of GDF15 and the risk of dementia, cerebrovascular disease, cognitive impairment, and brain atrophy, which are clinical features of many neurodegenerative diseases (Fuchs et al., 2013; Chai et al., 2016; Jiang et al., 2016; Nasrabady et al., 2018). In particular, some studies suggested that high circulating levels of GDF15 are associated with the risk of developing AD, as well as other neurodegenerative diseases, and considered this protein as a promising diagnostic biomarker and therapeutic target of several neurodegenerative diseases (Chai et al., 2016; Wu et al., 2021; Xue et al., 2022). On the other hand, other studies have found no difference in the circulating level of GDF15 in AD patients compared to age-matched healthy controls (Conte et al., 2021), or showed that exogenous recombinant GDF15 can promote Aβ clearance activity of microglial cultured cells (Kim et al., 2018). In addition, studies performed on *in vitro* and *in vivo* AD mice models demonstrated that the administration of recombinant GDF15 may exert beneficial effects by promoting the proliferation and migration of hippocampal stem cells, while GDF15 ablation leads to reduced proliferation and migration of these cells (Carrillo-Garcia et al., 2014; Kim et al., 2015). Therefore, the involvement of GDF15 in AD is likely more complex than expected, and it is still unclear whether GDF15 has detrimental or beneficial effects.

To date, most of the studies performed in humans regarding the possible association between GDF15, aging, and AD have been performed at circulating levels, while little is known about GDF15 levels in cerebrospinal fluid (CSF) and its expression and maturation in the human brain. The aim of this study is, therefore, to investigate the possible differences in CSF concentrations of GDF15 in AD patients with different degrees of disease severity, as well as its protein expression (both the precursor and the mature form, pro-GDF15 and m-GDF15 respectively) in different brain areas obtained from autoptic samples from subjects of different age (range 33–104 years) with or without AD.

## Materials and methods

### Samples

#### Cerebrospinal fluid (CSF) samples

Cerebrospinal fluid samples from 48 subjects in the age range 54–81 years were used. The subjects were admitted to the Alzheimer’s Center of the University of Milan, Fondazione IRCCS Ca′ Granda, Ospedale Policlinico, with suspicion of neurodegenerative dementias. The clinical workup included detailed past medical history, general and neurological examination, routine blood tests, formal neurocognitive assessment, brain computed tomography (CT) scan or magnetic resonance imaging (MRI), and, when indicated, [18F]-fludeoxyglucose positron emission tomography, as well as lumbar puncture (LP) for CSF biomarkers amyloid beta (Aβ-42), total Tau (T-Tau), and tau phosphorylated at position 181 (P-Tau181) determination. Normality references considered were Aβ-42 ≥ 600 pg./ml; T-tau ≤ 500 pg./ml for individuals older than 70 years and ≤ 450 pg./ml for individuals aged between 50 and 70 years; and P-tau 181 ≤ 61 pg./ml (Serpente et al., 2020). The diagnosis of AD was done according to current criteria (Dubois et al., 2014). In order to compare the data obtained from CSF with data obtained from plasma, as described in Conte et al. (2021), AD patients were selected from our previous study.

The study was approved by the local ethics committee (study n. 5,802 approved on 14-09-2021 by Comitato Etico Milano Area 2).

Samples were divided as follows: (i) 8 subjects diagnosed as non-AD with low CSF T-Tau level (T-Tau < 350 pg./ml) and mild cognitive impairment stable over at least 1–3 years of follow-up (age range 56–81); (ii) 20 AD patients with low CSF T-Tau level (T-Tau < 400 pg./ml; age range 54–81); and (iii) 20 AD patients with high CSF T-Tau level (T-Tau > 400 pg./ml; age range 55–81; see Table 1). For the control group (non-AD), we considered the eight individuals diagnosed as non-AD, since they were not cognitively impaired according to an mini-mental state examination (MMSE) test (MMSE ≥ 28), did not present alterations with instrumental analyses (CSF biomarkers, imaging) and did not develop dementia over a 1–3 year follow-up. Moreover, non-AD subjects showed significantly higher CSF levels of Aβ-42 with respect to AD patients, both high and low T-TAU (Table 1).

**Table 1:** Cerebrospinal fluid (CSF) sample description.

	Non-AD	AD with low T-Tau	AD with high T-Tau	Kruskal–Wallis test (*p*-values)
Number of subjects	8	20	20	
Age range (mean ± SD)	56–81 (73 ± 7.1)	54–81 (69.9 ± 8.4)	55–81 (70.2 ± 8.4)	
Sex (*N*)	4 M; 4 F	9 M; 11F	8 M; 12F	
Onset (*N*)	/	7 EOAD; 13 LOAD	10 EOAD; 10 LOAD	
T-TAU (mean ± SD pg/ml)	258.4 ± 70.3	297.2 ± 77.7	1115.8 ± 412.1	< 0.001
p-TAU (mean ± SD pg/ml)	51.8 ± 15.2	52.1 ± 9.7	108.4 ± 35.1	< 0.001
Aβ-42 (mean ± SD pg/ml)	1037.8 ± 299.5	476.3 ± 68.8	489.1 ± 89.5	< 0.001

EOAD, early onset Alzheimer’s disease; LOAD, late onset Alzheimer’s disease.

CSF samples were collected into 15 ml polypropylene tubes by lumbar puncture (LP) in the L3/L4 or L4/L5 interspace at about 8 and 10 a.m. after a one-night fast. Following LP, CSF samples were centrifuged at 2,000 rpm for 10 min at 4°C and the supernatants were aliquoted in polypropylene tubes and stored at −80°C until use. For each CSF sample Aβ-42, T-tau, and P-tau181 were measured using, respectively, three commercially available sandwich enzyme-linked immunosorbent assay (ELISA) kits: INNOTEST Amyloid-beta 42, tau, and P-tau181 assays (INNOTEST Fujirebio, Ghent, Belgium), conducted according to the instructions of the manufacturer.

#### Brain samples

Human autopsies of different brain areas from 32 subjects in the age range of 33–104 years were used. These samples were provided by: (1) the Abbiategrasso Brain Bank at the Golgi Cenci Foundation (Milan, Italy), and (2) the Immunology Lab at Bologna University (Responsible Prof. S. Salvioli).

1. Nineteen samples from the Abbiategrasso Brain Bank at the Golgi Cenci Foundation were divided into: (i) 6 non-demented old control subjects (NDO, age range 79–80 years) without evident signs of neurodegeneration, (ii) 12 AD patients (age range 75–89 years), (iii) 1 centenarian subject with age-related Tau-pathology (104 years). The study protocol received approval from the Ethical Committee of Pavia University (Committee report 3/2009).
2. Thirteen samples from the Immunology Lab at Bologna University were divided into: (i) 10 non-demented subjects, 3 adults in the age range 33–55 years (NDA) and 7 old in the age range 71–82 years (NDO), without evident signs of neurodegeneration, (ii) 1 patient with a diagnosis of AD (69 years), (iii) 2 centenarians affected by cognitive impairment who died of old age (103 years). All samples were collected in the framework of the European Project PROTEOMAGE (grant agreement: FP6-518230). The mean of the postmortem interval (PMI) for all subjects was 9.9 ± 4.5 h (range: 3–16 h).

ND subjects died from pathologies that did not affect the brain, and the older ones displayed no signs of cognitive impairment at death and presented only mild age-related neuropathological alterations. All samples categorized as AD came from patients with a clinical diagnosis of major neurocognitive disorder (major-NCD) according to DSM-5 (American Psychiatric Association, 2013) and a neuropathological diagnosis of AD. The post-mortem AD diagnosis was made according to the NIA-Alzheimer’s Association guidelines for the neuropathological assessment of AD, using the ABC score (a combination of Aβ plaques diffusion stage, Braak stage for TAUopathy, and CERAD semiquantitative grading for neuritic plaques; Montine et al., 2012). In particular, AD is defined by the concomitance of more severe pathology in both amyloid and Braak stages. A neuropathological characterization of samples was also carried out on formalin-fixed slices, embedded in paraffin. The sections were stained with hematoxylin and eosin, cresyl violet, luxol fast blue, and Gallyas to evaluate vascular, architectural, and structural tissue abnormalities, myelin loss, and neuritic plaques. For immunohistochemical analysis, NeuN and GFAP were used to evaluate neuronal and glial compartments, while AT8, 4G8, α-synuclein, and TDP43 antibodies were used to assess all the main proteinopathies (Poloni et al., 2020).

### Primary dermal fibroblasts (DFs) culture and *in vitro* GDF15 knock-down

Dermal fibroblasts (DFs) were obtained from biopsies of sun-protected areas from 11 non-demented subjects without neurodegenerative diseases (3 young subjects in the age range of 25–34 years and 8 old subjects in the age range of 73–78 years) and from 3 AD patients (age range 75–79 years). Dermal fibroblasts from control subjects were from Salvioli’s Lab in Bologna, whereas those from AD patients were from the Abbiategrasso Bank at the Golgi Cenci Foundation. In these latter subjects, the biopsy was performed at an average time of approximately 8 h post-mortem. Cells were cultured in DMEM-high glucose supplemented with 10% heat-inactivated fetal calf serum (FCS), penicillin (100 units/ml), streptomycin (100 mg/ml), and 2 mM L-glutamine (all from Sigma), in an incubator at 5% CO_2_, with a humidified atmosphere of 37°C. All the DFs used for the experiments were between the 5th and 12th passages.

Growth differentiation factor 15 knockdown was obtained through an RNA interference (RNAi) strategy. siRNA targeting human GDF15 and scramble negative control siRNA were provided by Cohesion Biosciences. A combination of two GDF15 siRNA was selected after testing the silencing efficacy of different combinations of three different siRNA targeting GDF15. The chosen combination in silencing efficacy was about 70%. Transfection was performed with ScreenFect siRNA reagent (ScreenFect GmbH), following the manufacturer’s instructions. Briefly, 125,000 cells were reseeded in 6-well plates, and scramble negative control siRNA or GDF15 siRNA was added to the cells with the transfection reagent. The medium with the siRNA was replaced after 24 h with a fresh complete medium. Cells were harvested after a further 72 h for RNA extraction.

### RNA extraction and gene expression analysis

RNA was available only from frontal cortex samples. Total RNA was isolated from about 50 mg of autoptic samples with the RNeasy Lipid Tissue Mini kit (Qiagen). The tissue was homogenized with the OMNI TH Tissue Homogenizer (OMNI international) in the lysis buffer supplied by the kit. RNA isolation was then performed following the manufacturer’s instructions. RNA concentration and purity were checked on a NanoDrop2000 spectrophotometer (Thermo Scientific), whereas RNA integrity was analyzed using a 2100 Bioanalyzer (Agilent Technologies). Samples with an RNA integrity number (RIN) ≥ 4 were included in the gene expression analysis.

Total RNA from DFs was isolated from cell pellets using the EasyPure RNA kit (TransGen Biotech Co., Ltd) according to the manufacturer’s instructions.

cDNA was synthetized using iScript^™^ cDNA Synthesis Kit (Bio-Rad) following the manufacturer’s protocol. Gene expression was analyzed by real-time RT-PCR, performed with iTaq^™^ Universal Sybr Green Supermix (Bio-Rad) and a Rotor gene Q 6000 system (Qiagen). Different housekeeping genes were tested for their stability as reference genes (*RNA18S1*, *ACTB*, ribosomal protein large P0, phospho-glycerate kinase 1 [*PGK1*], and glyceraldehyde-3-phosphate dehydrogenase [*GAPDH*]). *PGK1* and *GAPDH* were chosen as reference genes due to their more stable results and all data were normalized with respect to these genes. The relative expression ratio was then calculated using the 2^–ΔΔCT^ method. Expression analysis of the following genes was performed: *GDF15*, *ATF4*, *ATF3*, *DDIT3*, *TP53*, *IL6*, *NDUFA9*, *SDHA*, *UQCRC2*, *COX4I1*, *ATP5PD*. All predesigned primers were from Bio-Rad (primers information is available on the website).^1^

### Protein extraction and Western blotting analysis

Protein lysates were obtained from about 50 mg of frozen samples of different brain areas, with the exception of centenarians, from which only frontal cortex samples were available. A lysis buffer with the following composition was used: CHAPS 4%, Urea 8 M, DTT 65 mM, Tris 40 mM, phosphatase, and protease inhibitors (Sigma). Lysis was performed with the OMNI TH Tissue Homogenizer (OMNI international). The lysate was then centrifuged at 25000 rpm for 1 h at 4°C and the supernatant was collected. Total protein extract was quantified by Bradford’s method and stored at −80° until the analysis; 50 μg of protein extract were separated on a 12% or 16% SDS-polyacrylamide gel, transferred to a Polyvinylidene Difluoride (PVDF) or nitrocellulose membrane (Trans-Blot Transfer Medium, Bio-Rad) and then immunoblotted with primary antibodies (Table 2). To evaluate the content of respiratory chain complexes and ATP synthase, blots of resolved proteins were incubated with primary mouse/rabbit monoclonal antibodies specific for single subunits of each OXPHOS complex as reported in Barbato et al. (2015). As a loading control, GAPDH was used. Densitometry analysis of bands was performed using ImageJ software.

**Table 2:** List of primary antibodies used.

Primary antibody	Supplier	WB dilution
GAPDH	Novus Biological	1:20,000
GDF15/Mic-1	Cell Signaling	1:500
VDAC1	Abcam	1:2000
COX IV	Proteintech	1:1000
NDUFA9	Abcam	1:1000
SDHA	Abcam	1:2500
UQCRC2	Abcam	1:1000
ATP5H	Abcam	1:1000

### GDF15 ELISA

Quantikine ELISA Human GDF15 kit (R&D; minimum detectable dose 2.0 pg./ml) was used to evaluate GDF15 levels in cell culture supernatant and CSF, according to the manufacturer’s instructions. For both cell culture supernatant and CSF determinations, 50 μl per sample was used without dilution. For DFs supernatant determinations, cells were reseeded in 6-well plates (125,000 cells) and cultured for 72 h in supplemented DMEM. Then the supernatant was collected, centrifuged at 2500 rpm for 5 min, and frozen at −80°C until use. All samples were analyzed in duplicate, and the mean value was used for the statistical analysis. The standard curve was determined by analyzing simultaneously a dilution series of a standard sample. The intra- and inter-assay CV averages were 2.8 and 5.0%, respectively. Synergy^™^ fluorometer (Bio-Tek Instruments, Winooski, VT, United States) was used to read the absorbance of the plate.

### Immunofluorescence

The expression of different antigens such as Neuronal Nuclei (NeuN) and GDF15, TMEM119 and GDF15, as well as GDF15 and Glial Fibrillary Acidic Protein (GFAP), was investigated in control and AD tissues sections using a double sequential immunofluorescence procedure. Formalin-fixed paraffin-embedded sections were deparaffinized in xylene, rehydrated in ethanol at decreasing concentrations, and washed in distilled water. Then, tissue sections were pre-treated using microwave (4 cycles for 5 min each) and citrate buffer pH 6.0 for antigens retrieval method, cooled to room temperature (RT) for 15 min, and rinsed in PBS for 10 min. Afterward, slides were incubated with 1% bovine serum albumin (BSA) in PBS at RT for 30 min to block unspecific binding sites, probed with monoclonal NeuN (1:1,000; Chemicon, Millipore) or TMEM119 (1:500; Novus Biologicals) and polyclonal GDF15 (1:100; Cohesion Biosciences) primary antibodies and incubated at + 4°C overnight; polyclonal GFAP (1:500; Dako), was incubated at RT for 30 min. After washing with PBS, tissue sections were, respectively, labeled with Alexa Flour^™^ 488 anti-mouse (1:250; Invitrogen by Thermo Fisher Scientific) and Alexa Flour^™^ 546 anti-rabbit (1:250; Invitrogen by Thermo Fisher Scientific) secondary antibodies at 37°C for 1 h in the dark; all antibodies were diluted in 1% BSA in PBS, and all incubations were performed in a humidified chamber. For the detection of GFAP immunostaining, slides were stained with the Ready Probes Alexa Flour^™^ 488 anti-rabbit secondary antibody (Invitrogen by Thermo Fisher Scientific) at 37°C for 1 h in a humidified chamber in the dark. After rinsing in PBS, nuclei were counterstained with ProLong^™^ Gold antifade reagent with DAPI (Invitrogen by Thermo Fisher Scientific) and stored at + 4°C. Negative controls were obtained by processing sections without primary antibodies. Digital images were acquired using a Leica DMI6000 B inverted fluorescence microscope (Leica Microsystems, Wetzlar, Germany).

### Transmission electron microscopy

For the ultrastructural analysis, 125,000 cells were reseeded in 6-multi-well plates and scramble negative control siRNA or GDF15 siRNA were added to the cells with the transfection reagent. After 96 h of treatments, the cells were washed in PBS before being fixed in 2.5% buffered glutaraldehyde at RT for 20 min. From each well, cells were recovered by scraping and then transferred into microtubes before proceeding with centrifugation. The obtained cellular pellets were stored in the same fixative at + 4°C overnight. Then, samples were rinsed, post-fixed in 1% buffered osmium tetroxide for 1 h at RT, gradually dehydrated with ethanol through increasing concentrations, and embedded in Araldite resin. After sectioning samples with ultramicrotome, the ultrathin sections were counterstained with uranyl acetate and lead citrate and observed in a Philips CM100 (FEI Company, ThermoFisher, Waltham, MA, United States) Transmission Electron Microscope, and digital images were acquired with an Olympus camera.

### Thiobarbituric acid reactive substances analysis in frontal cortex samples

To measure the amount of lipid peroxidation in the frontal cortex samples, the following protocol was used. Briefly, 200 μl of 30% trichloroacetic acid (TCA) was added to 150 mg of homogenized samples, followed by the addition of 1.5 ml of phosphate buffer (pH 7) and 1.5 ml of 0.8% thiobarbituric acid (TBA). Samples were then incubated at 95°C for 20 min. After 10 min of cooling at 4°C, samples were centrifuged at 1,500 rpm for 10 min at 4°C. After centrifugation, the organic layer was taken and absorbance analysis was performed with 530 nm excitation.

### Statistical analysis

All tests used are reported in each figure legend. For normally distributed data, a Student’s *t*-test or one-way ANOVA was performed. Data that did not follow a normal distribution were analyzed by using Mann–Whitney or Kruskal–Wallis tests. Bonferroni correction was applied to correct multiple comparisons. The Association between mRNA expression of GDF15 and of the other genes analyzed was assessed with the Pearson correlation test and regression analysis. Correlation analyses of GDF15 levels in CSF and plasma, as well as m-GDF15 protein levels with age, were analyzed with the Spearman’s test, as data did not follow a normal distribution. *p* < 0.05 was considered as the accepted level to discriminate significant from non-significant results. Data are expressed as mean ± SE or SD. All analyses were performed using the software SPSS 23.0 for Windows.

## Results

### The CSF levels of GDF15 are similar in non-AD subjects and in AD patients with either low or high T-tau levels

**Figure 1:**
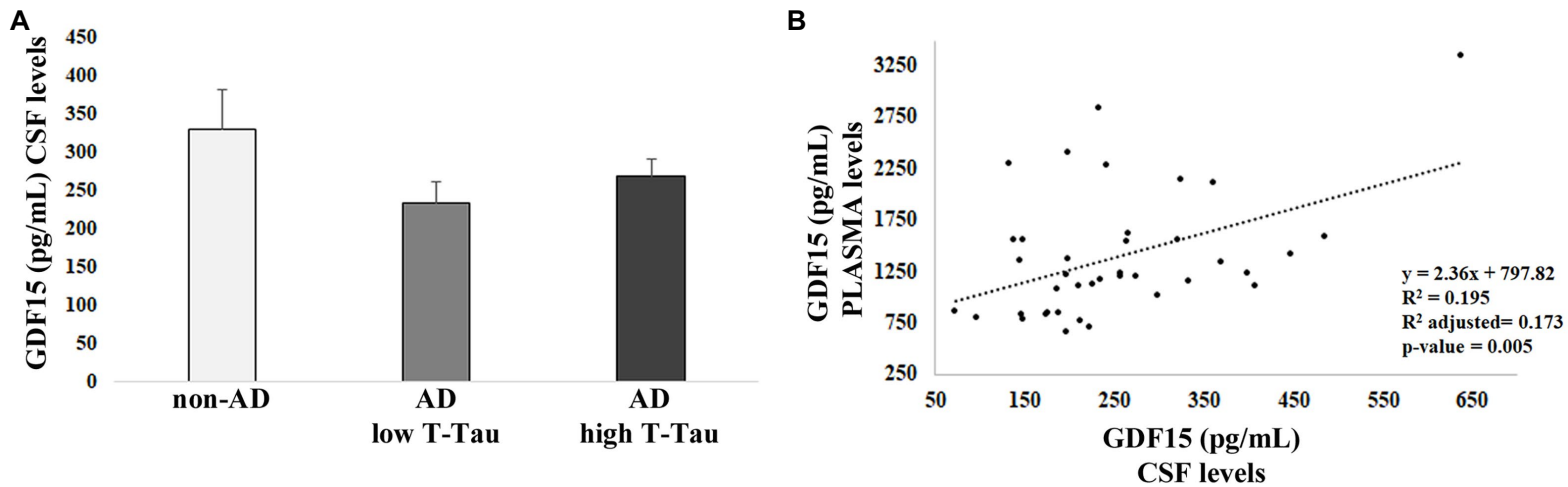
ELISA analysis of growth differentiation factor 15 (GDF15) levels in cerebrospinal fluid (CSF) and plasma. **(A)** GDF15 CSF level in 8 non-AD subjects (non-AD), 20 AD patients with low T-Tau CSF level [Alzheimer’s disease (AD) low T-Tau], and 20 AD patients with a high CSF T-Tau level (AD high T-Tau). **(B)** Regression analysis of GDF15 levels in CSF and plasma of the same subjects.

From our previous studies, no difference in plasma levels of GDF15 was found between AD patients and controls. However, we reasoned that possible differences could be found at the level of CSF. Therefore, we performed an ELISA test for GDF15 on the CSF of 40 AD patients (AD), subdivided into two groups on the basis of the levels of total Tau (T-Tau, considered as a biomarker for the degree of neuronal damage), as compared with the CSF of 8 age-matched, non-demented subjects (non-AD) with mild cognitive impairment (MCI) stable over at least 3 years. No difference was noticed between the three groups (Figure 1A), supporting our previous data indicating that circulating levels of GFD15 are not correlated with AD (Conte et al., 2021).

We then performed Spearman’s correlation and regression analysis that pointed out a positive correlation between GDF15 levels in plasma, as measured in Conte et al. (2021), and CSF of the same subjects (*ρ* = 0.378; *p* = 0.02; Figure 1B), suggesting a tight relationship between the levels of GDF15 in the two fluids.

### Growth differentiation factor 15 is expressed in the human brain and positively correlates with *TP53*, *ATF3*, and *IL-6*

Despite the lack of difference in the CSF levels of GDF15 between AD and non-AD, we wondered whether GDF15 could be differentially expressed in human brain samples obtained from AD and non-demented old subjects (NDO).

**Figure 2:**
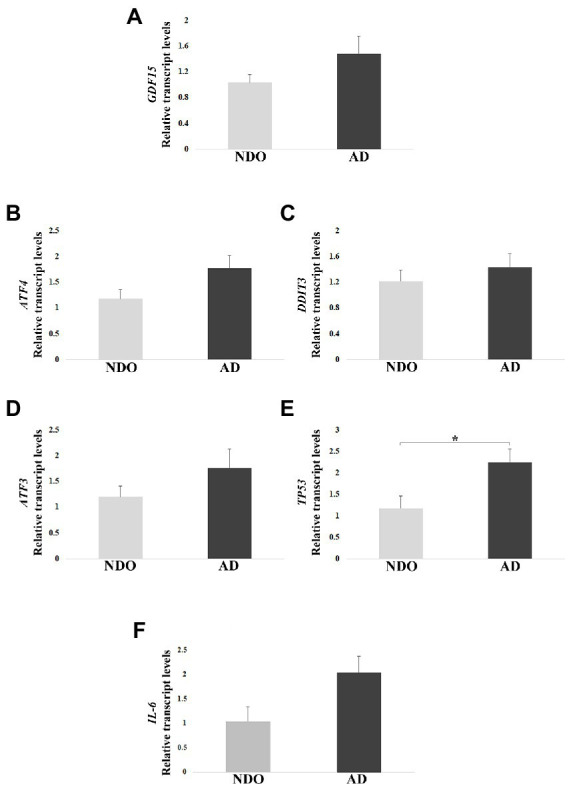
Real-Time RT-PCR analysis in AD patients (AD) and age-matched non-demented old controls (NDO). **(A)** GDF15, **(B)** ATF4, **(C)** DDIT3, **(D)** ATF3, **(E)** TP53, and **(F)** IL-6 relative transcript levels in frontal cortex samples from 10 AD and 5 NDO. The bars represent mean ± SE. Student’s *t* test was applied. ^*^p < 0.05.

We first performed a real-time RT-PCR analysis in frontal cortex samples from 10 AD and 5 NDO. *GDF15* transcript was expressed in the frontal cortex and its level appeared to be higher in AD with respect to NDO, though with no significant difference (Figure 2A). To further characterize these samples, we investigated the expression of the main GDF15 transcription factors, such as *ATF4*, *ATF3*, *DDIT3* and *TP53*, which for instance are also strongly implicated in AD (Hooper et al., 2007; Baleriola et al., 2014; Zhu et al., 2014; Wei et al., 2015). The expression of *ATF4*, *ATF3,* and *TP53* followed the same trend of *GDF15*, with a higher expression in AD than in NDO. However, only the transcript level of *TP53* showed significantly higher expression in AD compared to NDO (Figures 2B–E). In addition, we analyzed the transcript level of the pro-inflammatory cytokine interleukin 6 (*IL-6*), as AD is characterized by neuroinflammation (Leng and Edison, 2021) and considering that GDF15 acts as an anti-inflammatory molecule (Lambert et al., 2015; Abulizi et al., 2017; Chung et al., 2017; Moon et al., 2020; Conte et al., 2020a). Similar to the other analyzed genes, the *IL-6* transcript level followed the same trend of *GDF15*, with a higher expression in AD compared to NDO (Figure 2F).

In order to check possible associations among the transcript levels of all tested genes, we performed a Pearson correlation. A positive correlation of *GDF15* with *TP53*, *ATF3*, and *IL-6* expression was found, as well as among *IL-6*, *TP53*, and *ATF3* transcripts (Table 3). Moreover, p53 transcript levels positively correlated with *ATF3* and *DDIT3* (Table 3).

**Table 3:** Pearson correlation analysis among mRNA levels of tested genes in frontal cortex from 10 Alzheimer’s disease (AD) and 5 non-demented old subjects (NDO).

		*GDF15*	*IL-6*	*TP53*	*ATF3*	*ATF4*	*DDIT3*
*GDF15*	r	1					
	p						
*IL-6*	r	0.680^**^	1				
	p	0.005					
*TP53*	r	0.728^**^	0.645^*^	1			
	p	0.003	0.013				
*ATF3*	r	0.614^*^	0.605^*^	0.542^*^	1		
	p	0.015	0.017	0.045			
*ATF4*	r	0.061	0.096	0.359	0.229	1	
	p	0.830	0.734	0.208	0.412		
*DDIT3*	r	0.389	0.076	0.698^**^	0.387	0.477	1
	p	0.152	0.788	0.006	0.154	0.072	

^*^p < 0.05; ^**^p < 0.01.

### GDF15 protein is processed more in AD and centenarians and seems to be predominantly expressed by neurons

**Figure 3:**
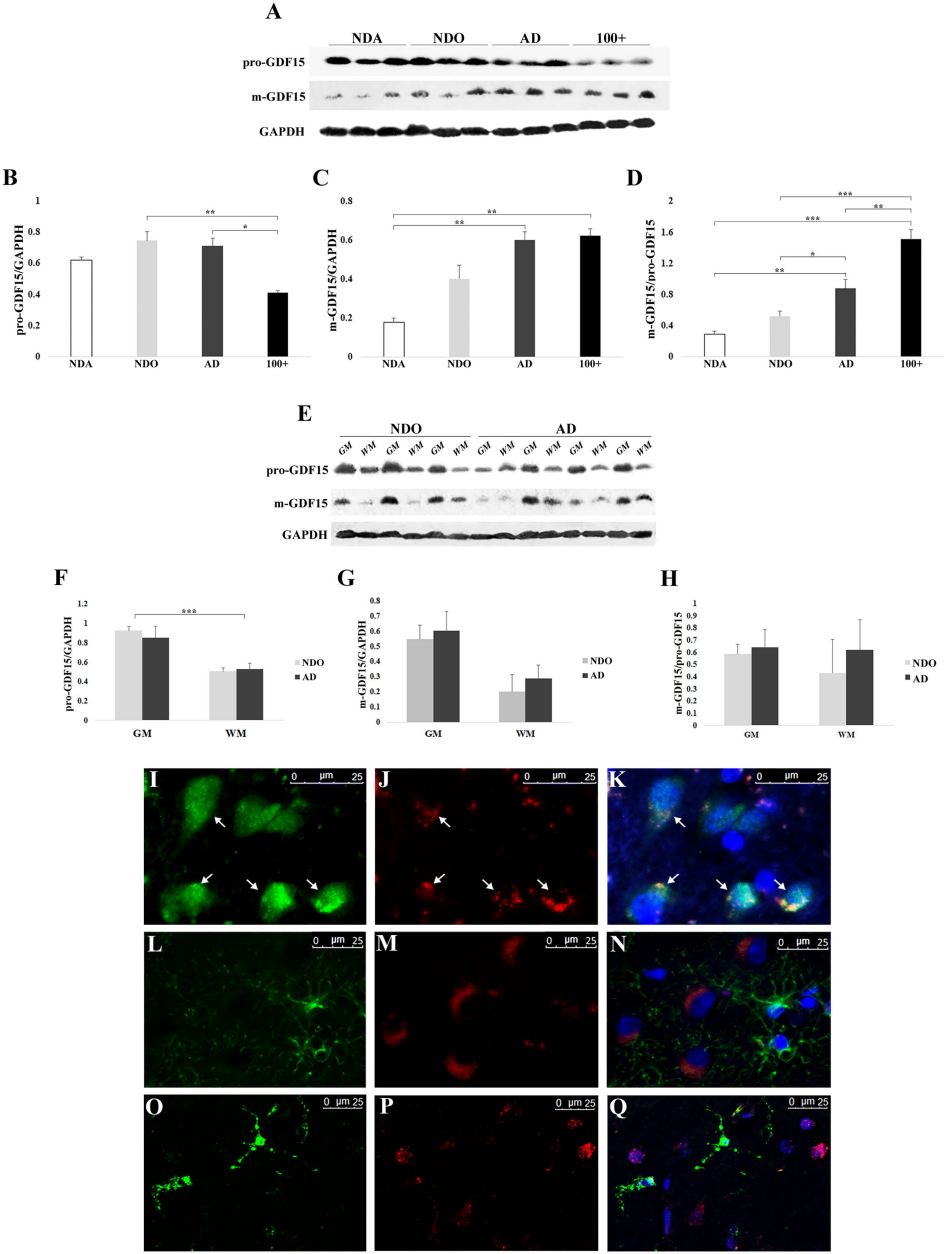
Western blotting and immunofluorescence analyses of GDF15 in the frontal cortex. **(A)** Representative immunoblotting image of pro-GDF15, m-GDF15, and GAPDH in frontal cortex. Relative protein expression of **(B)** pro-GDF15, **(C)** m-GDF15, and **(D)** m-GDF15/pro-GDF15 ratio in frontal cortex from three non-demented adults (NDA), 11 non-demented old subjects (NDO), 11 AD patients (AD) and three centenarians (100+). **(E)** Immunoblotting image of pro-GDF15 and m-GDF15 in gray (GM) and white (WM) matter of frontal cortex from 3 NDO and 4 AD. Relative protein expression of **(F)** pro-GDF15, **(G)** m-GDF15 and **(H)** m-GDF15/pro-GDF15 in GM ad WM from 3 NDO and 4 AD. **(I-Q)** Fluorescence microscopy analysis in frontal cortex. **(I)** NeuN (green), **(J)** GDF15 (red), and **(K)** merge; nuclei (DAPI). Arrows indicate colocalization of GDF15 and NeuN signals. **(L)** GFAP (green), **(M)** GDF15 (red), and **(N)** merge; nuclei (DAPI). **(O)** TMEM119 (green), **(P)** GDF15 (red), and **(Q)** merge; nuclei (DAPI). The bars represent mean ± SE. Student’s t and one-way ANOVA tests with Bonferroni correction were applied. Western blotting quantification was performed using ImageJ software and normalized to GAPDH expression. ^*^p < 0.05. ^**^p < 0.01. ^***^p < 0.001.

We extended our analysis by evaluating the effect of age and AD on the expression of the precursor (pro-GDF15) and mature (m-GDF15) forms of GDF15 protein in frontal cortex samples. To this aim, we took advantage of samples obtained not only from NDO and AD but also from non-demented adults aged 33–55 years (NDA) and centenarians (100+). Pro-GDF15 expression was higher in NDO and AD compared to 100+ (Figures 3A,B), while that of m-GDF15 was higher in AD and 100 + compared to NDA (Figures 3A,C). In order to have an indication of the rate of GDF15 processing, we calculated the m-GDF15/pro-GDF15 ratio. This ratio was higher in AD and 100 + compared to NDA and NDO, and also in 100 + compared to AD (Figure 3D). Interestingly, a significant positive correlation between age and m-GDF15 (*r* = 0.63, *p* = 0.003) but not pro-GDF15 was also present in the brain, similar to that found at circulating levels, confirming previous studies indicating that m-GDF15 is one of the most up-regulated proteins during aging (Tanaka et al., 2018; Conte et al., 2019; Lehallier et al., 2019; Conte et al., 2020b). To further characterize GDF15 expression patterns in the frontal cortex, we separately evaluated the gray and white matter of the same NDO and AD subjects. Both pro-GDF15 and m-GDF15 were expressed more in gray matter than white matter (Figures 3E–H). Moreover, in order to assess whether the expression of GDF15 was limited to specific cell types, we performed in the same samples a double immunofluorescence labeling with antibodies specific for neuronal (NeuN), astrocytes (GFAP), or microglia (TMEM119) markers and GDF15. The fluorescence microscopy analysis indicated that GDF15 seems to be predominantly expressed by NeuN + cells, but not GFAP + or TMEM119 + ones (Figures 3I–Q).

As samples were available from other areas of the brain, we further extended our investigation by checking possible differences in GDF15 expression levels among different brain areas (frontal cortex, hippocampus, temporal cortex, parietal cortex, and cerebellum) separately in both NDO and AD. The levels of both pro-GDF15 and m-GDF15 were largely similar among areas in NDO and AD samples (Supplementary Figures S1A–G). However, for AD only, the m-GDF15/pro-GDF15 ratio was reduced in the parietal cortex with respect to the hippocampus (Supplementary Figure S1G).

**Figure 4:**
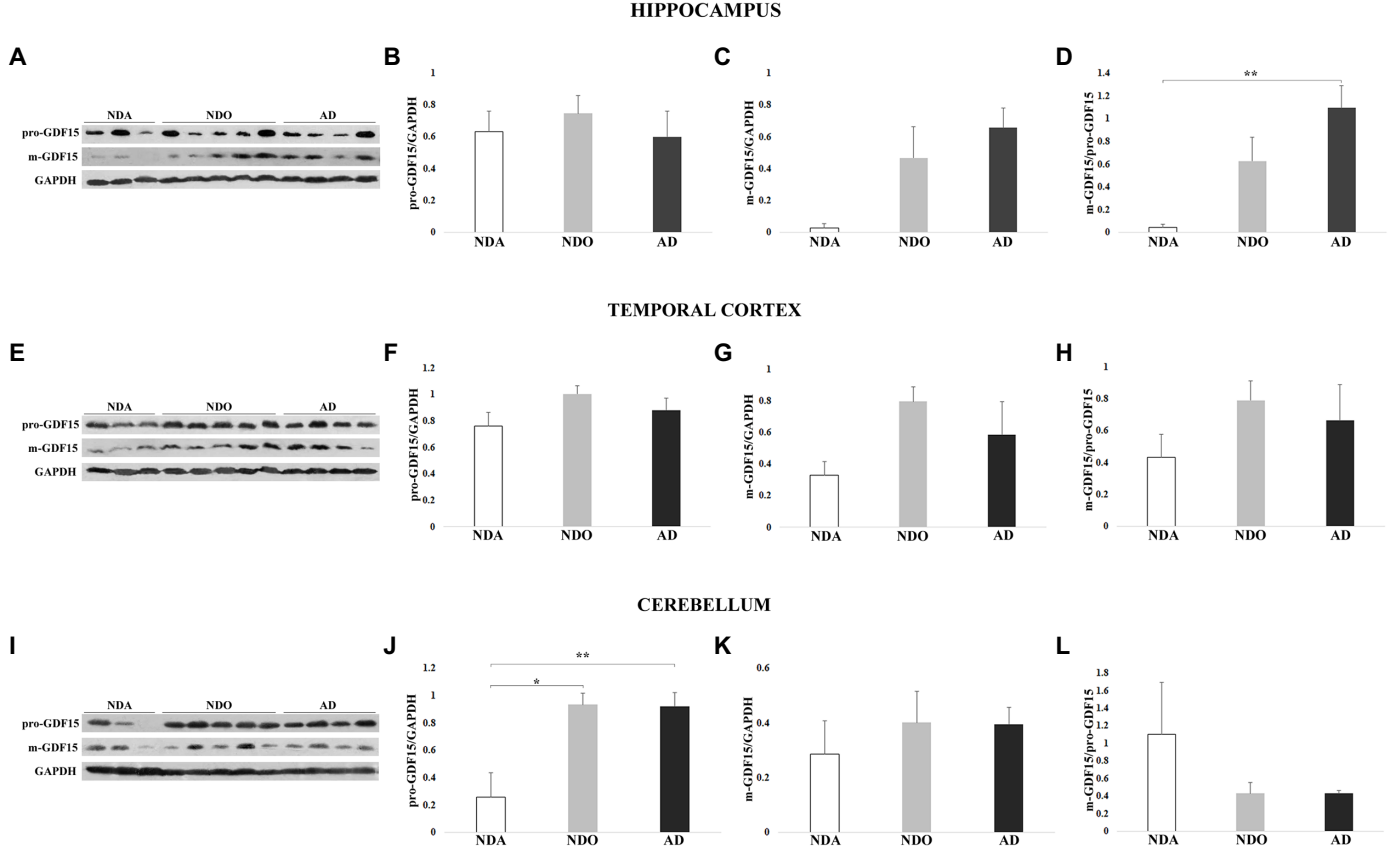
Western blotting analysis of GDF15 in different brain areas from 3 non-demented adults (NDA), 5 non-demented old subjects (NDO), and 4 AD patients (AD). **(A)** Representative immunoblotting image and **(B)** pro-GDF15, **(C)** m-GDF15, and **(D)** m-GDF15/pro-GDF15 ratio relative protein expression in hippocampus, **(E–H)** temporal cortex, and **(I–L)** cerebellum. The bars represent mean ± SE. Student’s t and one-way ANOVA tests with Bonferroni correction were applied for hippocampus and temporal cortex analyses. Kruskal–Wallis test and Bonferroni correction were applied for cerebellum analysis. Western blotting quantification was performed using ImageJ software and normalized to GAPDH expression. ^*^p < 0.05. ^**^p < 0.01.

We then sought differences in the levels of GDF15 between different groups (NDA, NDO, and AD) within the same areas. In the hippocampus, pro-GDF15 was expressed similarly in all groups considered, while m-GDF15 expression tended to be higher in NDO and AD compared to NDA. Interestingly, the m-GDF15/pro-GDF15 ratio was significantly higher in AD compared to NDA (Figures 4A–D), similar to what we found in the frontal cortex. Also in the temporal cortex, m-GDF15 and the m-GDF15/pro-GDF15 ratio seem to be higher in NDO and AD with respect to NDA but without statistical differences (Figures 4E–H). In the cerebellum, pro-GDF15 expression was significantly higher in NDO and AD with respect to NDA, while no differences were found for m-GDF15. Furthermore, no significant differences were found for the m-GDF15/pro-GDF15 ratio (Figures 4I–L). In the parietal cortex, for which only samples from NDO and AD were available, no differences in the expression of pro-GDF15 and m-GDF15 were observed (Supplementary Figure S2).

Overall, these findings indicate that GDF15 is predominantly expressed by neurons and that this expression is modulated by the presence of AD and, possibly, by extreme aging. Moreover, it appears that this modulation occurs especially in the frontal cortex and hippocampus but not the parietal, temporal cortex, and cerebellum.

### Expression of mitochondrial complexes I, III, and V is lower in frontal cortex samples from AD with respect to NDO

**Figure 5:**
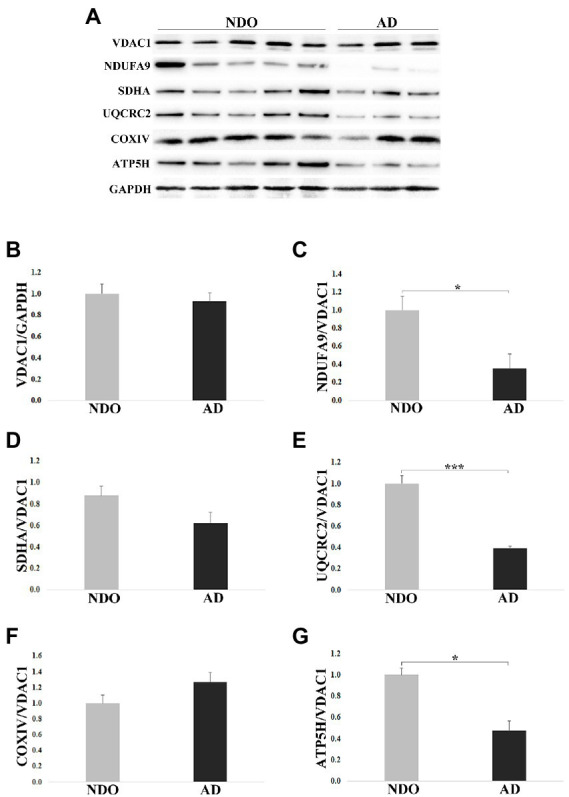
Western blotting analysis of mitochondrial complex subunits in frontal cortex from 11 non-demented old subjects NDO and 6 AD patients (AD). **(A)** Representative immunoblotting image, **(B–G)** relative protein expression of VDAC1, NDUFA9, SDHA, UQCRC2, COXIV and ATP5H. The bars represent mean ± SE. Student’s *t* and one-way ANOVA tests with Bonferroni correction were applied. Western blotting quantification was performed using ImageJ software and normalized to GAPDH expression. Relative expression of OXPHOS proteins was normalized to VDAC1 expression. ^*^p < 0.05. ^***^p < 0.001.

As AD samples were characterized by higher levels of m-GDF15 and p53 (a redox-sensitive protein) with respect to NDO, we checked whether AD samples were also characterized by elevated oxidative stress. To this aim, we measured the levels of thiobarbituric acid reactive substances (TBARS), which can be used to assess the level of oxidative stress in biological samples (Aguilar Diaz De Leon and Borges, 2020), in frontal cortex samples from 4 NDO and 5 AD. No differences were found between NDO and AD (NDO: 1.214 ± 0.418 μM/mg of tissue; AD: 1.513 ± 0.506 μM/mg of tissue). However, as briefly mentioned before, GDF15 is associated with mitochondrial dysfunction and has been recently proposed as a biomarker for mitochondrial diseases (Montero et al., 2016; Poulsen et al., 2020). Thus, we characterized in frontal cortex samples from 11 NDO and 6 AD the protein expression of representative subunits of the mitochondrial complexes (Figures 5A–G). In order to evaluate the mitochondrial mass and normalize the expression of the complexes’ subunits, we also analyzed the protein expression of VDAC1. VDAC1 expression was similar in NDO and AD, suggesting the presence of a similar mitochondrial mass in both groups (Figure 5B). NDUFA9 (Complex I; Figure 5C), UQCRC2 (Complex III; Figure 5E) and ATP5H (Complex V; Figure 5G) expression was reduced in AD compared to NDO. Finally, SDHA (Complex II; Figure 5D) and COXIV (Complex IV) expression (Figure 5F) was similar in both groups.

Taken together, these data suggest that elevation of GDF15 expression occurs together with an alteration in the abundance of OXPHOS complexes, even though, due to the low number of samples, the analysis of correlation was not statistically significant (data not shown).

### *In vitro* modulation of GDF15 expression affects mitochondrial gene expression and morphology, and inflammatory marker

As it is not possible to establish a cause-effect relationship between GDF15 and mitochondrial dysfunction in *ex vivo* fixed samples, we exploited an *in vitro* cell model where GDF15 modulation was feasible. In particular, we made use of DFs from AD patients as compared to ND subjects. In fact, different alterations observed in the brain of AD patients have often been observed in other tissues, leading to the hypothesis that AD could be considered a systemic disease. In particular, DFs are considered a reliable model to study metabolic and mitochondrial alterations typical of AD (Uberti et al., 2002; Pérez et al., 2017). Therefore, we took advantage of DFs cultures from 11 ND (3 from young subjects, age range 25–34 years, and 8 from old subjects, age range 73–78 years) and 3 AD patients (age range 75–79 years) to evaluate the levels of GDF15 and the effects of its modulation on IL-6 and mitochondrial complexes expression.

**Figure 6:**
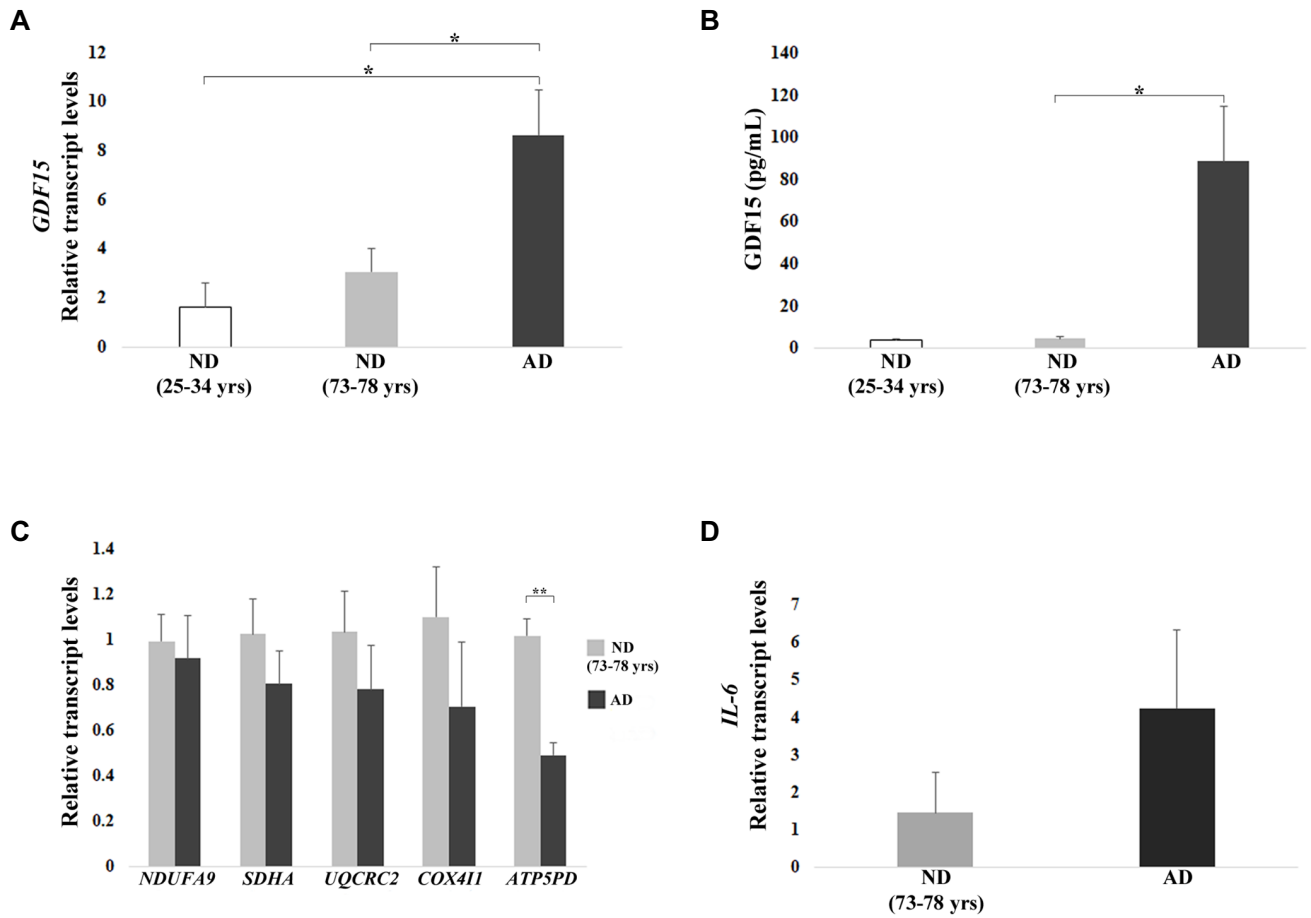
Real-time RT-PCR analyses and ELISA in dermal fibroblasts (DFs) from three non-demented young subjects (ND, age range 25–34 years), 8 non-demented old subjects (ND, age range 73–78 years), and 3 AD patients (AD). **(A)** Relative *GDF15* transcript level and **(B)** quantification by ELISA of GDF15 protein secreted in the culture medium. **(C)** Mitochondrial complex subunits (*NDUFA9*, *SDHA*, *UQCRC2*, *COX4I1*, *ATP5PD*) and **(D)** *IL-6* relative transcript levels. The bars represent mean ± SE. Student’s *t* and one-way ANOVA tests with Bonferroni correction were applied for transcript-level analyses. Kruskal–Wallis test was applied for ELISA analysis [AD vs. ND (25–34 years) *p* = 0.067]. ^*^p < 0.05. ^**^p < 0.01.

First, we analyzed the transcript level of *GDF15* in DFs, which was significantly higher in AD DFs as compared to ND DFs from both young and old subjects (Figure 6A). To confirm this finding, we analyzed the level of the GDF15 protein released in the culture medium. Secreted GDF15 levels showed even more dramatic differences, being much higher in AD DFs. In particular, secreted GDF15 levels were significantly higher in AD DFs compared to ND (73–78 years), whereas, due to experimental variability, the statistical significance was borderline when comparing AD and ND (25–34 years) DFs (Kruskal–Wallis test *p* = 0.067; Figure 6B).

We then investigated the transcript levels of representative subunits of the mitochondrial complexes in order to test whether also GDF15 could be associated with mitochondrial dysfunction in DFs. We observed a trend of lower expression for all subunits in AD with respect to ND (73–78 years), except for *ATP5PD*, which was significantly lower (*p* < 0.01) in AD as compared with ND (73–78 years; Figure 6C). Moreover, given the positive correlation between *GDF15* and *IL-6* levels observed in frontal cortex samples, we also analyzed the expression levels of *IL-6*, which tended to be higher in AD DFs compared to ND (73–78 years; Figure 6D).

**Figure 7:**
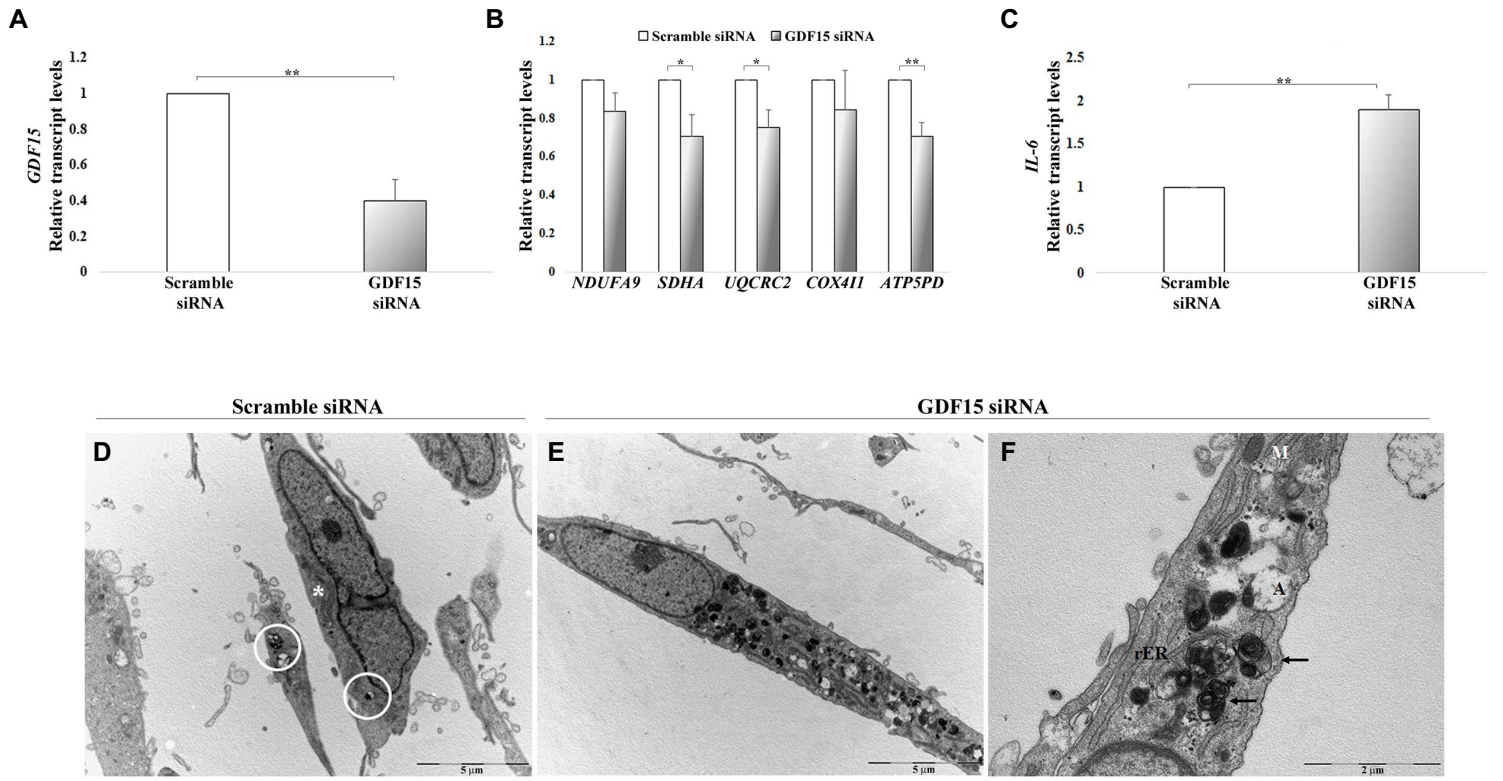
GDF15 knock-down (KD) in dermal fibroblasts (DFs). **(A–C)** Real-time RT-PCR analysis in DFs from fives non-demented old subjects in the age range 73–78 (ND) and 3 AD patients (AD) after GDF15 KD by small interfering RNA (siRNA) technique. Relative transcript levels of **(A)** *GDF15*, **(B)** mitochondrial complex subunits (*NDUFA9*, *SDHA*, *UQCRC2*, *COX4I1*, *ATP5PD*), and **(C)** *IL-6* in scramble siRNA and GDF15 siRNA DFs. The bars represent mean ± SE. Student’s *t* and one-way ANOVA tests with Bonferroni correction were applied for transcript-level analyses. ^*^p < 0.05. ^**^p < 0.01. **(D–F)** Transmission electron microscopy. The ultrastructural morphology of DFs has been compared in scramble siRNA-treated DFs and GDF15 siRNA-treated DFs. **(D)** Scramble siRNA-treated DFs show a spindled morphology with long bidirectional projections. In the cytoplasm there are several mitochondria (*) some of which are degenerated (white circle). Scale bar = 5 μm. **(E)** After GDF15 KD (GDF15 siRNA), DFs accumulate degenerated mitochondria that appear as electron-dense round bodies associated with autophagosomes. Scale bar = 5 μm. **(F)** Magnification of panel E showing degenerated dense mitochondria with whorled cristae (arrows) and autophagosomes. Scale bar = 2 μm. Legend: M: normal mitochondrion; rER: rough endoplasmic reticulum; A: autophagosomes.

We then performed a gene knock-down (KD) in DFs from both ND (73–78 years) and AD with a siRNA targeting GDF15, and we analyzed the mRNA level of mitochondrial complex subunits and IL-6. A consistent silencing efficacy was obtained (Supplementary Figure S3A). As both ND and AD DFs KD for GDF15 showed similar results, the data were then pooled together (Figures 7A–C). GDF15 KD induced a significant decrease in transcript levels of complex II, III, and complex V subunits, and a significantly higher expression of IL-6 compared to scramble siRNA (Figures 7B,C). Moreover, since an alteration in OXPHOS complexes expression was found, in order to investigate possible changes in mitochondria morphology, we also performed a transmission electron microscopy analysis. Interestingly, GDF15 siRNA-treated DFs are characterized by a higher number of degenerated mitochondria appearing as electron-dense round bodies associated with whorled cristae and autophagosomes (Figures 7D–F). These results are in favor of the idea that GDF15 has anti-inflammatory and mitochondria-protective effects, and that its elevated expression in AD brain samples is to be considered an attempt to counteract an ongoing mitochondrial stress.

## Discussion

Alzheimer’s disease (AD) is a devastating neurodegenerative disease for which effective therapy is still not available and current pharmaceutical approaches are largely unsatisfactory since at best they can delay the progression of the symptoms (Gauthier et al., 2016). This is possibly due to the fact that current drugs act on downstream events and cannot avoid neuron loss (Mehta et al., 2017; Yiannopoulou et al., 2019). Therefore, the attention of researchers has turned toward other more upstream phenomena, including reduced glucose utilization and insulin resistance (Kellar and Craft, 2020), altered autophagy and loss of proteostasis (Morawe et al., 2012), increased oxidative stress and inflammation (Wyss-Coray and Rogers, 2012), and mitochondrial dysfunction (Selfridge et al., 2013). In this complex scenario, mitochondria seem to play a central role, as they are at the crossroad between many, if not all, of these phenomena. Accordingly, a large amount of evidence has shown that mitochondria impairment precedes the clinical onset of AD, indicating the critical role of these organelles in the development of this neuropathology (Swerdlow, 2018; Wang et al., 2020). In particular, it has been demonstrated that enhancing mitochondrial proteostasis by targeting mitochondrial translation or mitophagy can reduce amyloid aggregation (Sorrentino et al., 2017). Mitochondrial dysfunction is now emerging not only as a source of reactive oxygen species (ROS), which are tightly linked to inflammation, but also as a trigger for a number of stress responses aimed at restoring cellular homeostasis, including UPR^mt^ (Shen et al., 2022). UPR^mt^ and mitophagy are considered important quality control mechanisms and accordingly, the dysregulation of UPR^mt^-related proteins leads to neuronal decline during aging (Rugarli and Langer, 2012). In AD, a transcriptional upregulation of genes involved in UPR^mt^ and mitophagy has been reported (Beck et al., 2016; Sorrentino et al., 2017) and it has been interpreted as a protective response during disease progression (Mufson et al., 2012). Among the key regulators of UPR^mt^ are ATF4, and DDIT3, as well as ATF3 (see Shen et al., 2022 for references). These transcription factors, together with others including p53, promote the expression of GDF15. In particular, literature data indicate that ATF3 acts as a co-regulator of p53 in the expression of GDF15, and loss of either p53 or ATF3 severely alters GDF15 mRNA levels (Catizone et al., 2020). Despite these data indirectly suggesting the involvement of GDF15 in AD, there is a substantial lack of consensus on whether GDF15 is actually a player in this neurodegenerative disorder. In this study, we observed that: (i) GDF15 is expressed and processed in human brain areas; (ii) GDF15 appears to be predominantly expressed by neurons; (iii) in some areas (frontal cortex and hippocampus) the processing into the mature form seems to be higher for AD patients with respect to age-matched non-demented subjects; (iv) accordingly, a lower expression level of representative OXPHOS proteins was found in AD samples with respect to non-demented subjects; v. the expression of *GDF15* appears strongly associated with *IL-6* expression. Finally, and contrary to expectations, CSF levels of GDF15 are not different between AD patients and non-AD subjects, but are strongly associated with plasma GDF15 levels. This was the first step of the present work, as previous studies have found an association between the circulating levels of GDF15 and the risk of dementia, cerebrovascular disease, cognitive impairment as well as brain atrophy, and AD (Fuchs et al., 2013; Chai et al., 2016; Jiang et al., 2016; Nasrabady et al., 2018; Wu et al., 2021; Xue et al., 2022). We were not able to confirm these data in a previous study that analyzed the plasma of 120 AD patients as compared with 194 age-matched controls including 102 offspring of centenarians, who are considered to be a model of successful aging (Conte et al., 2021). We reasoned that maybe the amount of GDF15 produced by the brain could be too small to influence the plasma concentration, which is likely more affected by other organs, such as the bladder, kidney, prostate, liver, and muscles (Wischhusen et al., 2020; Conte et al., 2022), but it could still be possible to find differences in the CSF. However, this hypothesis is inconsistent with our results, as the levels of GDF15 in the CSF are similar among non-AD subjects and AD patients with either low or high levels of T-Tau. Interestingly, the CSF levels of GDF15 are positively correlated with the plasmatic ones, suggesting also that, for CSF, the levels of GDF15 are dictated by the production occurring in organs other than the brain. In this regard, it should be considered that in the elderly the permeability of both blood–brain and blood-CSF barriers rises, with an increase in the plasmatic influence on CSF composition (Erickson and Banks, 2019).

Despite these results on CSF, GDF15 results are not only expressed in human brain areas, but also its modification into the mature form (which is considered the main secreted one) is higher in samples from AD with respect to NDA and age-matched controls, suggesting that stress is likely occurring in these patients that elicits a chronic elevation in GDF15 expression. Interestingly enough, m-GDF15 and m-GDF15/pro-GDF15 ratios are even higher in samples from centenarians who did not show profound cognitive impairment nor neuropathological features comparable to AD. This suggests that the stress-response machinery is well preserved in these exceptional individuals and that GDF15 is by no means detrimental, as these individuals delayed the onset of age-related diseases such as AD by many years or even decades. Therefore, high levels of GDF15 in AD do not likely represent a cause of the disease, but rather an attempt (in this case unsuccessful) to cope with it. It is not clear why the differences between groups were evident only in the frontal cortex and (partially) hippocampus but not in other areas affected by the disease such as the temporal and parietal cortices. We reasoned that maybe this was because of a sort of “saturation” of GDF15 expression in these latter areas that could not allow the detection of differences. However, this seemed not to be the case, as the levels of GDF15 expression appeared similar within the different areas investigated. Further investigations are needed to clarify this point.

Mitochondrial dysfunction seems to be a hallmark of AD (Selfridge et al., 2013). Accordingly, we have observed a lower expression of proteins belonging to respiration complex I, III, and V in AD samples as compared to NDO samples, despite the presence of a similar mitochondrial mass, thus suggesting an impairment of the mitochondrial respiratory function in AD brains. Surprisingly, alterations in Complex IV reported in the literature (Holper et al., 2019) were not confirmed, probably due to the low number of samples. Even though we were not able to directly document oxidative stress, the elevated expression of the redox-sensitive *TP53* transcription factor and the positive correlation of *GDF15* with *IL-6* expression are suggestive of an inflammatory response to mitochondrial dysfunction in which GDF15 is likely part of a network aimed at modulating this response. In fact, considering that GDF15 has anti-inflammatory activity (Lambert et al., 2015; Abulizi et al., 2017; Chung et al., 2017; Moon et al., 2020; Conte et al., 2020a), the association between GDF15 and IL-6 could be interpreted as an attempt of brain cells to compensate stress and restore homeostasis. In further support of this conclusion, in *in vitro* experiments, we have found that the temporary abrogation of GDF15 in DFs led to an increase in *IL-6* expression, a decrease in mitochondrial complexes, as well as an alteration in mitochondrial morphology.

This study has some limitations, mainly due to the limited availability of human brain samples, which did not allow the analyses in all areas for all groups considered. Finally, other questions remain open, such as the expression and regulation of proteases that cleave the precursor form of GDF15 into the mature form in the brain. To date, proprotein convertase, subtilisin/kexin-type (PCSK) 3, 5, and 6 have been recognized as proteases able to cleave pro-GDF15 in *in vitro* and *in vivo* studies on cardiomyocytes, heart, and prostate cancer (Couture et al., 2017; Li et al., 2018). However, it is not clear if and how much these proteins are expressed in the brain or whether other proteases may be needed.

**Figure 8:**
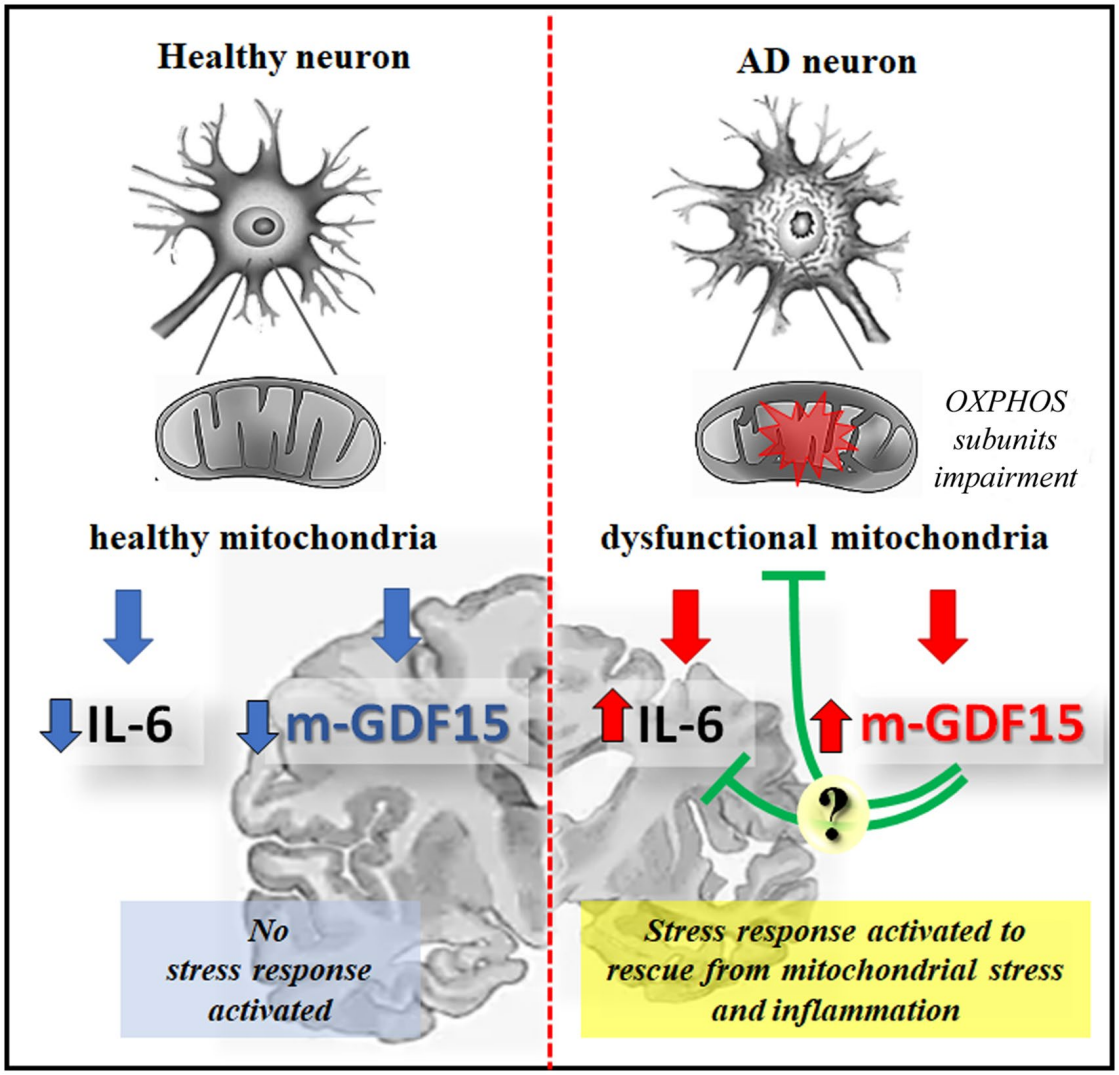
Schematic representation of GDF15 expression in healthy or AD neurons. In healthy aging, functional mitochondria are associated with a low level of inflammatory cytokines, such as IL-6, and of the mature form of GDF15 (m-GDF15), and no stress response is activated; in AD neurons an impairment of OXPHOS subunits expression is associated to mitochondrial dysfunction leading to a stress response that is associated to an increase of IL-6 and m-GDF15 protein expression. The elevated level of m-GDF15 in AD brains could be interpreted as a part of a stress response aimed at counteracting inflammation and mitochondrial stress.

In conclusion, while in our research, GDF15 appears not to be a reliable circulating marker of AD, it is nevertheless expressed in brain areas, and the processing into the mature form is higher in AD samples, where there is also an impairment of OXPHOS subunits expression, possibly indicating an attempt of the cells to rescue from a mitochondrial stress, as illustrated in Figure 8. Further studies could clarify whether GDF15 is always beneficial but (in some cases) not sufficient to rescue cells from stress, and what is the difference (if any) between AD patients and centenarians in terms of response efficacy. Several studies in mouse models demonstrated that chronic activation of the integrated stress response (ISR), another retrograde stress response sharing many transcription factors with UPR^mt^ and GDF15, can cause cognitive disorders (Krukowski et al., 2020). The suppression of ISR activation alleviates AD symptoms (Ma et al., 2013; Hwang et al., 2017), therefore, we cannot totally exclude the possibility, though unlikely, that the chronic expression of GDF15 may play a role in the pathogenesis of AD and thus GDF15 could be considered as a potential target to treat AD.

## Data availability statement

The original contributions presented in the study are included in the article/Supplementary material, further inquiries can be directed to the corresponding author.

## Ethics statement

The studies involving human participants were reviewed and approved by study n. 5,802 approved on 14-09-2021 by Comitato Etico Milano Area 2 the Ethical Committee of Pavia University (Committee report 3/2009). The patients/participants provided their written informed consent to participate in this study.

## Author contributions

AC: data generation and collection, statistical analysis, writing of the manuscript. SV, GP: fluorescence microscopy, transmission electron microscopy analysis, manuscript revision. AB, GSg, GSo: analysis of mitochondrial complexes, manuscript revision. VM, VF, TP, DG, MA: samples and sample data provision, manuscript revision. MT: TBARS analysis. CF, MCa: critical discussion of the manuscript. SS, MCo: study design, analysis of the data, writing of the manuscript. All authors contributed to the article and approved the submitted version.

## Funding

The study was partially supported by the Roberto and Cornelia Pallotti Legacy for Cancer Research to SS and by Centro Dino Ferrari and Fondazione Gigi & Pupa Ferrari to DG.

## Conflict of interest

The authors declare that the research was conducted in the absence of any commercial or financial relationships that could be construed as a potential conflict of interest.

## Publisher’s note

All claims expressed in this article are solely those of the authors and do not necessarily represent those of their affiliated organizations, or those of the publisher, the editors and the reviewers. Any product that may be evaluated in this article, or claim that may be made by its manufacturer, is not guaranteed or endorsed by the publisher.

## Footnotes

^1^ www.bio-rad.com/PrimePCR

## Supplementary material

SUPPLEMENTARY FIGURE S1 **(A)** Representative immunoblotting image of pro-GDF15, m-GDF15, and GAPDH in the frontal cortex (Fr), hippocampus (Hi), temporal cortex (Te), parietal cortex (Pa) and cerebellum (Ce). **(B–G)** Relative protein expression levels of pro-GDF15, m-GDF15, and m-GDF15/pro-GDF15 ratio from **(B–D)** 2 non-demented old subjects (NDO) and **(E–G)** 4 AD patients (AD). The bars represent mean ± SE. Student’s *t* and one-way ANOVA tests with Bonferroni correction were applied. Western blotting quantification was performed using ImageJ software and normalized to GAPDH expression. ^*^*p* < 0.05.

SUPPLEMENTARY FIGURE S2 **(A)** Representative immunoblotting image of pro-GDF15, m-GDF15, and GAPDH in the parietal cortex. **(B)** pro-GDF15 and (C) m-GDF15 protein relative expression in the parietal cortex from seven non-demented old subjects (NDO) and 11 AD patients (AD). The bars represent mean ± SE. Student’s *t* test was applied. Western blotting quantification was performed using ImageJ software and normalized to GAPDH expression.

SUPPLEMENTARY FIGURE S3 Relative transcript levels of **(A)** *GDF15* and **(B)** *IL-6* in DFs from five non-demented old subjects in the age range 73–78 (ND) and 3 AD patients (AD), considered separately, treated with scramble siRNA or GDF15 siRNA. The bars represent mean ± SE. Student’s *t* and one-way ANOVA tests with Bonferroni correction were applied.
