# Supplementary figures and images for: The expression pattern of GDF15 in human brain changes during aging and in Alzheimer’s disease
Source: Front Aging Neurosci. 2023 Jan 9;14:1058665. doi: 10.3389/fnagi.2022.1058665 (PMC9869280; doi:10.3389/fnagi.2022.1058665)

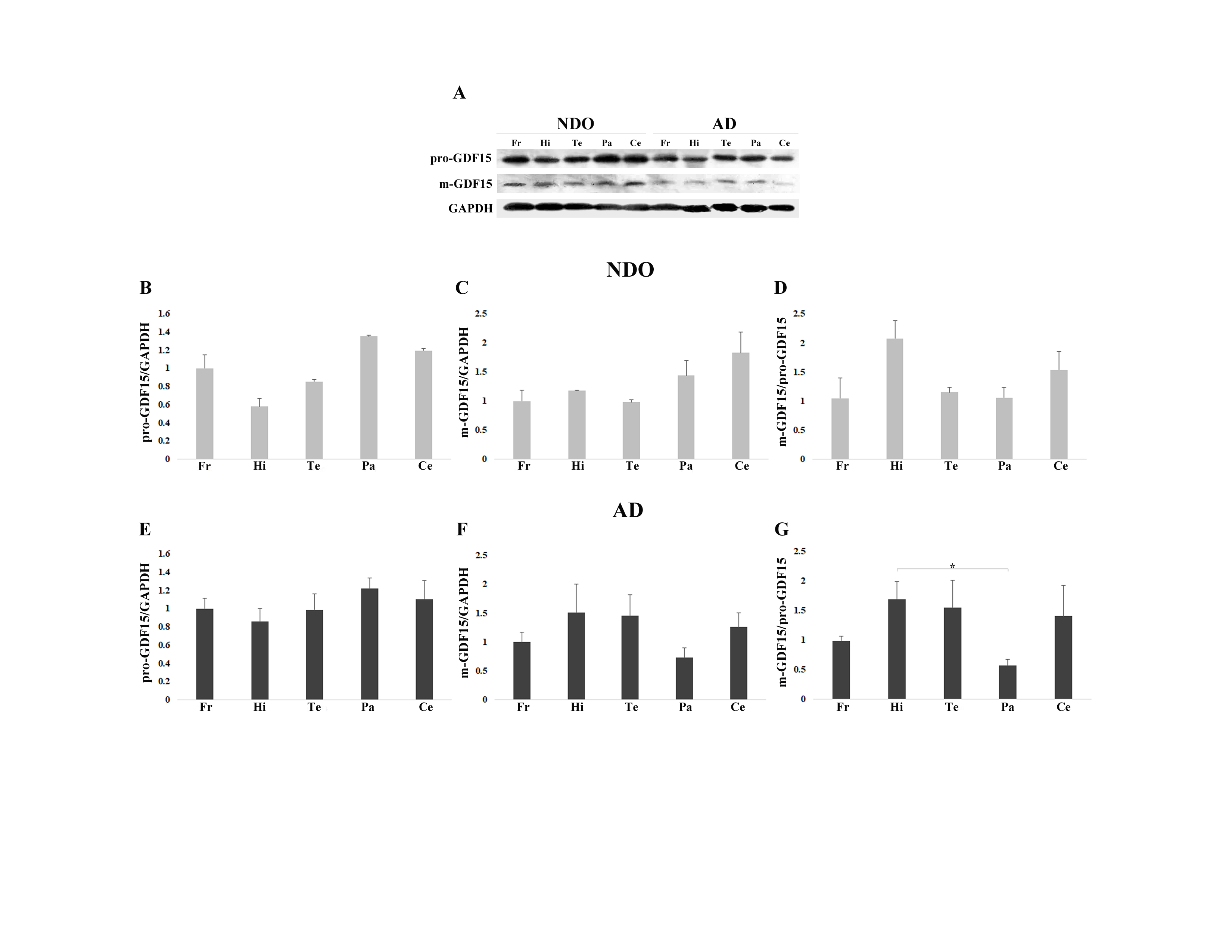

Supplement: SUPPLEMENTARY FIGURE S1 — (A) Representative immunoblotting image of pro-GDF15, m-GDF15, and GAPDH in the frontal cortex (Fr), hippocampus (Hi), temporal cortex (Te), parietal cortex (Pa) and cerebellum (Ce). (B–G) Relative protein expression levels of pro-GDF15, m-GDF15, and m-GDF15/pro-GDF15 ratio from (B–D) 2 non-demented old subjects (NDO) and (E–G) 4 AD patients (AD). The bars represent mean ± SE. Student’s t and one-way ANOVA tests with Bonferroni correction were applied. Western blotting quantification was performed using ImageJ software and normalized to GAPDH expression. *p < 0.05. [file Image_1.TIF]

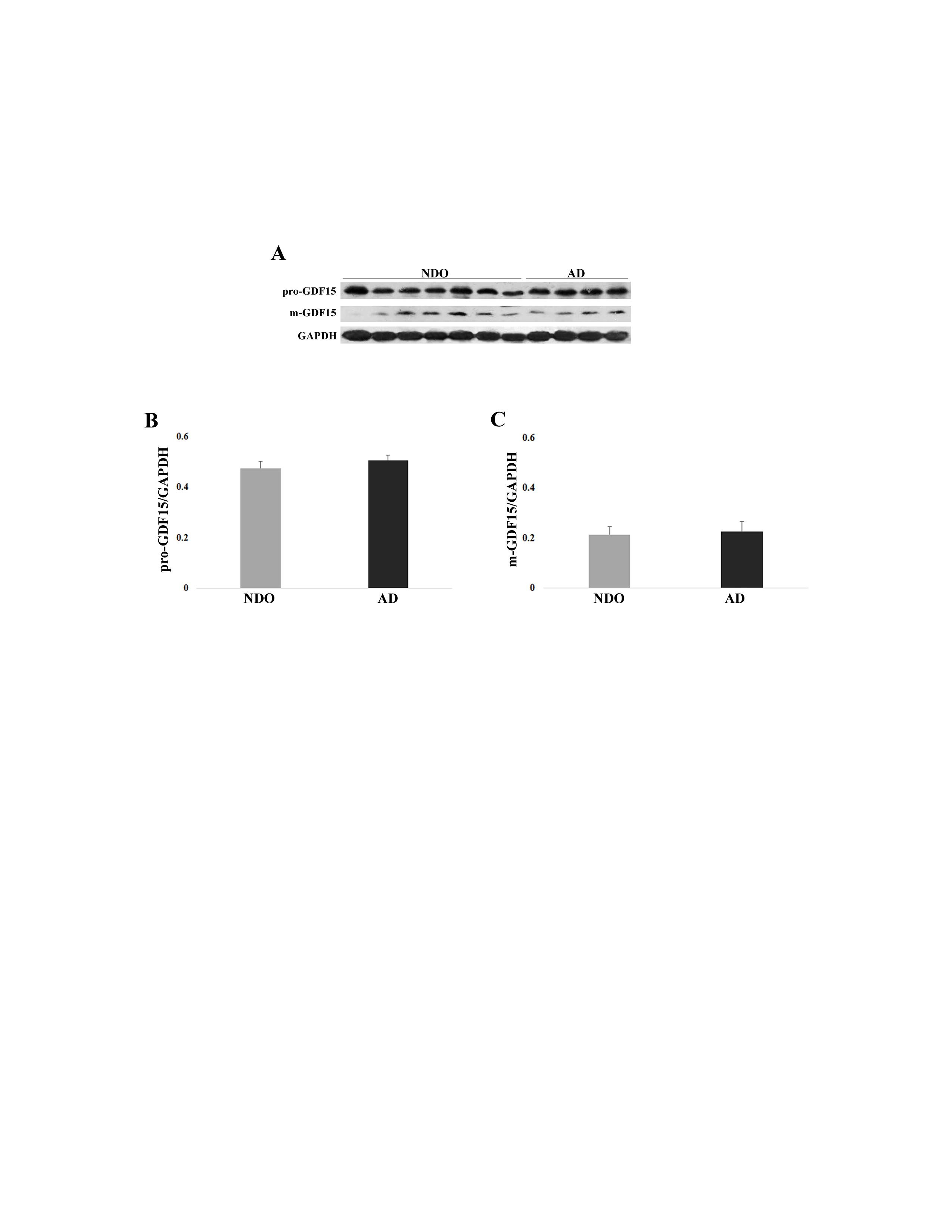

Supplement: SUPPLEMENTARY FIGURE S2 — (A) Representative immunoblotting image of pro-GDF15, m-GDF15, and GAPDH in the parietal cortex. (B) pro-GDF15 and (C) m-GDF15 protein relative expression in the parietal cortex from seven non-demented old subjects (NDO) and 11 AD patients (AD). The bars represent mean ± SE. Student’s t test was applied. Western blotting quantification was performed using ImageJ software and normalized to GAPDH expression. [file Image_2.TIF]

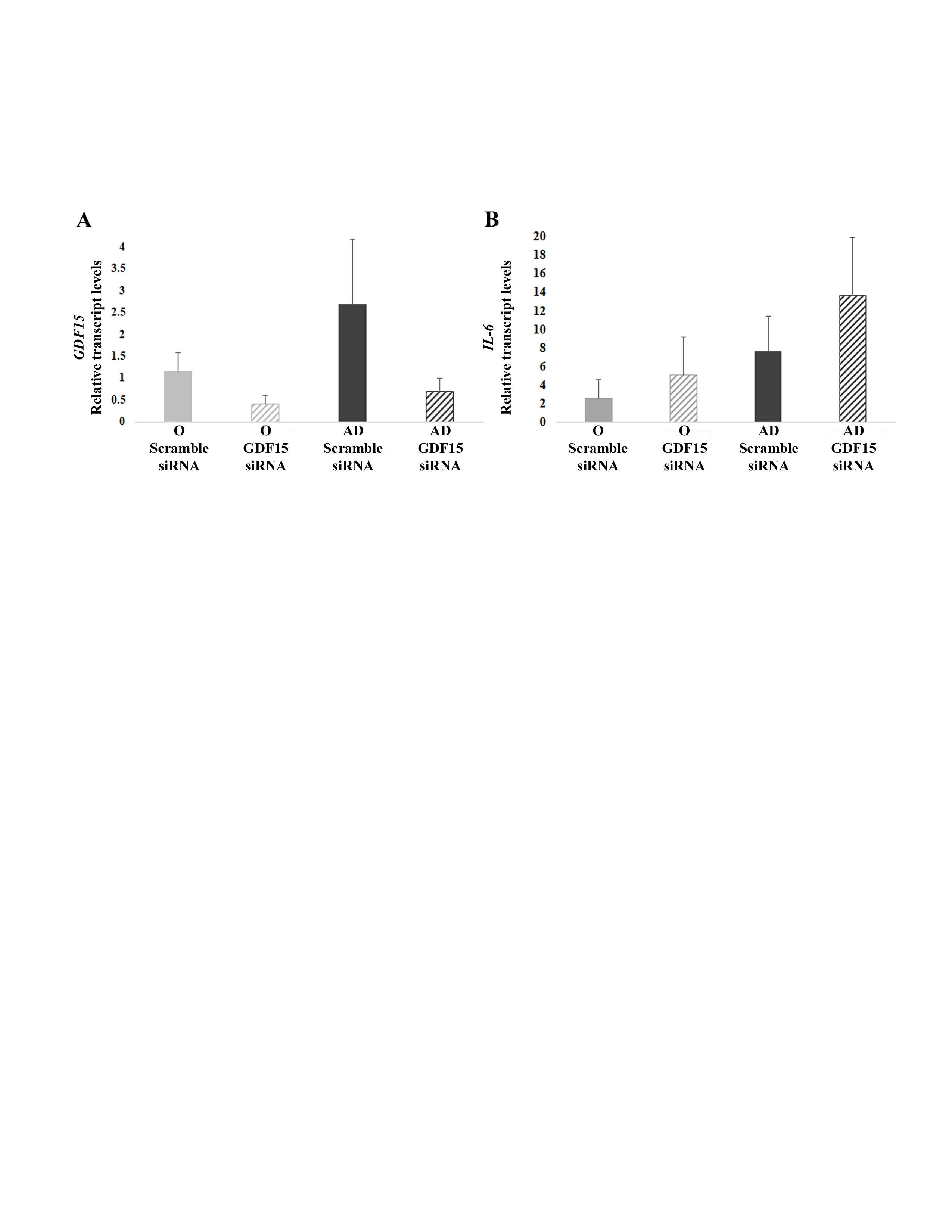

Supplement: SUPPLEMENTARY FIGURE S3 — Relative transcript levels of (A) GDF15 and (B) IL-6 in DFs from five non-demented old subjects in the age range 73–78 (ND) and 3 AD patients (AD), considered separately, treated with scramble siRNA or GDF15 siRNA. The bars represent mean ± SE. Student’s t and one-way ANOVA tests with Bonferroni correction were applied. [file Image_3.TIF]
